# Available Treatments for Autism Spectrum Disorder: From Old Strategies to New Options

**DOI:** 10.3390/ph18030324

**Published:** 2025-02-25

**Authors:** Liliana Dell’Osso, Chiara Bonelli, Federico Giovannoni, Francesca Poli, Leonardo Anastasio, Gianluca Cerofolini, Benedetta Nardi, Ivan Mirko Cremone, Stefano Pini, Barbara Carpita

**Affiliations:** Department of Clinical and Experimental Medicine, University of Pisa, 67 Via Roma, 56126 Pisa, Italy; liliana.dellosso@unipi.it (L.D.); f.giovannoni10@gmail.com (F.G.); f.poli22@studenti.unipi.it (F.P.); l.anastasio1@studenti.unipi.it (L.A.); gianlucacerofolini@gmail.com (G.C.); benedetta.nardi@live.it (B.N.); ivan.cremone@gmail.com (I.M.C.); stefano.pini@unipi.it (S.P.); barbara.carpita1986@gmail.com (B.C.)

**Keywords:** ASD, pharmacological treatments, new options

## Abstract

Autism spectrum disorder (ASD) is a condition that is gaining increasing interest in research and clinical fields. Due to the improvement of screening programs and diagnostic procedures, an increasing number of cases are reaching clinical attention. Despite this, the available pharmacological options for treating ASD-related symptoms are still very limited, and while a wide number of studies are focused on children or adolescents, there is a need to increase research about the treatment of ASD in adult subjects. Given this framework, this work aims to review the available literature about pharmacological treatments for ASD, from older strategies to possible new therapeutic targets for this condition, which are often poorly responsive to available resources. The literature, besides confirming the efficacy of the approved drugs for ASD, shows a lack of adequate research for several psychopharmacological treatments despite possible promising results that need to be further investigated.

## 1. Introduction

Autism spectrum disorder (ASD) is a neurodevelopmental disorder with a clinical picture characterized by a persistent deficit in socio-emotional reciprocity, communication, and interaction, as well as narrow and restricted interests and behavioral patterns. Despite its early onset, forms without intellectual or language impairment may remain underdetected, with subjects reaching clinical attention during adulthood due to symptoms related to other comorbid mental disorders [1,2,3]. Although males typically report higher rates of ASD diagnosis, several studies have stressed the importance of sex differences in ASD manifestations, highlighting the presence of female-specific features, which may result in an underdiagnosis of ASD among females [1]. Moreover, the tendency to mask dysfunctional behaviors in favor of socially acceptable ones, also known as camouflaging, is more pervasive and widespread in autistic female subjects, leading to a greater misdiagnosis of the disorder [1]. According to a dimensional approach to psychopathology [4,5,6,7], ASD clinical manifestations could be considered the tip of an iceberg, while sub-threshold autistic-like manifestations of variable kind and severity, known as autistic traits (ATs), would be distributed along a continuum, from the general population to a clinical sample where they became extremely marked [1]. The importance of ATs has been variously stressed in the literature: in this framework, sub-clinical and atypical manifestations, as well as isolated signs and symptoms, symptom clusters, and behavioral patterns, have also been hypothesized to represent vulnerability factors for developing different types of clinical pictures along an illness trajectory that would start from an autistic-like neurodevelopmental alteration [1,4,5,6,7,8,9].

During the last few decades, several studies have focused on the possible pathophysiological mechanisms underlying ASD [10]. When characterized by heterogeneous features, different factors seem to be involved in ASD pathogenesis, described as a result of the combination of genetic and non-genetic causes [10]. The presence of mild ATs in first-degree relatives of ASD subjects suggests a significant genetic component in the genesis of this disorder [11]. To date, more than 200 genes have been identified as ASD susceptibility genes [12,13,14] with monozygotic twin concordance ranging from 73% to 95%, estimated heritability over 90%, and a notable sibling recurrence risk of 5–6% for non-syndromic ASD [15]. A growing field of literature focuses on the role of the environment, especially the intrauterine one, considering advanced maternal and paternal age, valproate intake, toxic chemical exposure, maternal diabetes, and enhanced steroidogenic activity among potential risk factors for ASD [16,17]. Robust research investigated the presence of different kinds of biochemical correlates: among them, an enhanced inflammatory activity and microbiota alteration was highlighted in ASD subjects, which could be linked to an immune system and/or gut–brain axis dysfunction [18,19]. Indeed, immune dysregulation during pregnancy and the postpartum period may interfere with brain connection development and function [19]. The serotonin (5-HT) system is also considered to be involved in ASD pathogenesis, as ASD subjects generally report increased 5-HT levels relative to their neurotypical peers, possibly due to abnormalities in serotoninergic synthesis [20,21]. Moreover, tryptophan metabolic routes, including the kynurenine pathway, seem to be altered in ASD subjects [18]. Additional studies have highlighted disruption in glutamate excitatory and GABA inhibitory systems, resulting in the hyper-excitability of ASD cortical circuits [22,23]. Furthermore, lower levels of oxytocin, a neuropeptide involved in social bonding and recognition, may be linked to ASD deficits in social interactions [24]. Noticeably, recent studies have highlighted the role of other biochemical correlates, such as the altered transmethylation metabolism of sulfur-containing amino acids, including homocysteine, which may signal altered methylation activity [25,26,27]. Circulating brain-derived neurotrophic (BDNF) levels have been reported to be altered in ASD subjects [25,26,27], a feature that could be eventually linked to the altered connectivity in ASD brains reported from neuroimaging studies [28] (see Table 1).

On the other hand, although several studies have reported the efficacy of different pharmacological therapies for the improvement of specific symptoms, there is still no uniformity of psychopharmacological indications for ASD treatment [29,30,31,32,33,34,35,36,37,38,39,40,41].

Given this framework, our review aims to review the state-of-the-art of pharmacological treatments for ASD.

## 2. Methods

From 1 March 2024 to 1 April 2024, a search was conducted on the PubMed and Google Scholar electronic databases with the following terms: “pharmacological treatments” or “pharmacotherapy” or “drugs” and “autism spectrum disorder (ASD)”, “innovative treatments” or “new therapeutic strategies” and “ASD”, and “nonpharmacological therapies for ASD”. For each section, we selected related articles from the past 30 years, paying more attention to the more recent ones. We divided pharmacological treatments into six categories: antipsychotics, mood stabilizers, antidepressants, psychostimulants, alpha-2 adrenergic receptor agonists, cholinesterase inhibitors, and NMDA-receptor antagonists. Concerning innovative treatments and new strategies, we focused on oxytocin, anti-inflammatory and immunomodulatory drugs, bumetanide, sulforaphane, balovaptan, pioglitazone, and galantamine, and microbiota-transfer therapy. Finally, we also dedicated a section to nonpharmacological approaches to ASD.

## 3. Pharmacological Treatment for Autism Spectrum Disorders (ASD)

ASD pharmacological treatment is mainly focused on treating specific symptoms rather than the disorder itself. During recent decades, several studies have evaluated the use of different classes of drugs, like antipsychotics, mood stabilizers, antidepressants, psychostimulants, alpha-2 adrenergic receptor agonists, cholinesterase inhibitors, and NMDA-receptor antagonists [42].

### 3.1. Antipsychotics

Antipsychotic drugs act on different brain areas by modulating the action of dopaminergic, serotonergic, and other receptor subtypes with variable affinities. These drugs have been widely used to treat schizophrenia and other psychotic disorders [43], disruptive behaviors, and other symptoms in individuals with ASD. To date, risperidone and aripiprazole are the only two FDA-approved drugs for the treatment of symptoms in ASD [44,45,46,47]. Moreover, a recent review proved risperidone and aripiprazole’s efficacy for the short-term treatment of emotional dysregulation and irritability in ASD [48]. However, there are several other drugs belonging to the antipsychotics category that have been studied for ASD treatment, including quetiapine, ziprasidone, olanzapine, haloperidol, pimozide, paliperidone, clozapine, and lurasidone [49,50,51,52,53]. At the same time, the literature from recent decades has focused on the potential physiological effects that antipsychotics may have during the treatment of the disorder, aiming to find specific strategies for autistic patients [54].

#### 3.1.1. Typical Antipsychotics

##### Haloperidol

In children and adolescents, haloperidol has proven efficacy in short-term treatment for chronic symptoms associated with ASD. A first study, conducted on 40 children with ASD aged over 4 years and treated with haloperidol (dose 0.5 to 4.0 mg/day), demonstrated that the drug was significantly superior to placebo in reducing the severity of social withdrawal and stereotypes in this population [55]. Subsequent studies have confirmed these data, also highlighting good effectiveness in reducing maladaptive behaviors, irritability, emotional lability, feelings of anger, and poor collaboration, and in improving learning and psychosocial environment [56,57]. Higher age, higher IQ, and the presence of more severe symptoms seem to be predictive of a better response to haloperidol [58]. As expected, adverse effects, including sedation, dyskinesias, acute dystonia, and akathisia, were present [56,57,58]. Moreover, haloperidol was reported to be less effective than atypical antipsychotics [59,60].

##### Pimozide

The use of pimozide in patients with ASD is still controversial due to the limited studies available in the literature. In a double-blind, placebo-controlled study, 87 subjects with ASD, aged between 3 and 16 years, were treated with pimozide with dosages ranging between 1 and 9 mg/day. In this study, the drug proved to be superior to placebo in the treatment of sleep and excretion disorders but not in the management of behavioral ones, thus proving to be quite ineffective [60]. Furthermore, acute dystonia and sedation have been reported following treatment with pimozide [61].

#### 3.1.2. Atypical Antipsychotics

##### Risperidone

Risperidone has been the most investigated and effective drug for the treatment of irritability cluster symptoms associated with ASD, such as outbursts of anger, aggression, and self-harm [44,45,62,63]. Its efficacy has been established in two large, randomized trials [44,45]. In 1998, McDougle et al. conducted a double-blind randomized study lasting 12 weeks on 31 adult subjects diagnosed with ASD. Risperidone at an average dose of 2.9 mg/day significantly reduced repetitive behavior, aggression, anxiety, nervousness, depression, and irritability in adult patients with ASD [62]. Furthermore, risperidone was well tolerated in the absence of extrapyramidal effects, cardiac events, or seizures [62]. This study, therefore, cleared the way to the next two larger randomized trials that led to the validation of the drug by the FDA in 2006. Both children and adolescents with ASD, aged 5–17 years, received risperidone with dosages ranging from 1.2 to 1.8 mg/kg for 8 weeks. Data reported a reduction of irritability, aggression, social withdrawal, repetitive behaviors, and hyperactivity [44,45]. The major adverse effects are increased appetite, weight gain, fatigue, drowsiness, dizziness, increased levels of prolactin (PRL), and the presence of extrapyramidal symptoms (EPS) [44,45]. Conversely, no alterations in the lipid or glucose profile were found despite being usually associated with the use of most atypical antipsychotics in other clinical populations [44,45]. Further studies on risperidone in children with ASD and with disruptive behavior supported short-term response rates ranging from 57% to 72% [64,65,66]. Finally, other studies have highlighted the importance of smaller risperidone doses in autistic children between 2 and 9 years (0.5–1.5 mg/day) to improve long-term outcomes, thus underlining the importance of early intervention [31,67].

##### Aripiprazole

In 2009, aripiprazole became the second drug approved by the FDA for the treatment of disruptive behaviors in ASD children aged 6–17 years [68]. This decision was based on the positive results of two randomized double-blind studies evaluating the efficacy and the safety of the drug in these subjects, who were randomized to receive either a placebo or aripiprazole with a discontinuation rate ranging between 10.3 and 10.6%. Treatment with aripiprazole also showed adverse effects such as sedation, drowsiness, increased appetite, weight gain, hypersalivation, and the presence of extrapyramidal symptoms (EPS) [46,47]. However, as expected by the partial agonist action of this drug, mean serum prolactin levels decreased from baseline to endpoint [46,47], and there were no significant prolongations of the QTc interval or other electrocardiogram anomalies [69]. To the best of our knowledge, only a few studies are comparing the efficacy and adverse effects of risperidone and aripiprazole that show no statistically significant difference between the two drugs during a two-month study [70,71,72].

##### Quetiapine

Studies conducted on quetiapine for the treatment of patients with ASD have unfortunately been inconclusive. In particular, two open-label studies were performed on a small number of ASD patients, reporting poor clinical efficacy with a response rate of <25% and a high incidence of side effects such as sedation, increased aggression, agitation, and weight gain [73,74]. At the same time, a growing field of literature reported cases with a higher response rate (40–60%) but burdened with the adverse effects described above [75,76]. Finally, quetiapine could be useful for sleep disorders and aggression, although a minor efficacy with respect to risperidone and aripiprazole is globally suggested in managing ASD symptoms [52,77].

##### Ziprasidone

No definitive studies have yet been performed that have led to the approval of ziprasidone in the treatment of patients with ASD. A first open-label study on 12 subjects with ASD, aged between 8 and 20, evaluated the efficacy, safety, and tolerability of the drug with dosages ranging between 20 and 120 mg/day. The authors reported a significant improvement in aggression, agitation, and irritability after 14 weeks of treatment. The drug was well tolerated, and some patients even lost weight [78]. Further research was carried out to evaluate the effects of switching to ziprasidone in 10 adult patients with ASD who had experienced excessive weight gain while taking risperidone or quetiapine. All 10 patients involved lost significant weight after 6 months of treatment with ziprasidone at an average dosage of 128 mg/day, maintaining good control of behavioral symptoms [79]. Other open studies or case reports have shown promising results for this treatment [52,80,81,82].

##### Olanzapine

Several studies have been carried out to evaluate the effectiveness of olanzapine in patients who have ASD, most of which, however, are open studies with conflicting data. While few trials have revealed olanzapine efficacy in treating ASD symptoms such as irritability, anger, anxiety, hyperactivity, social withdrawal, and use of language [83] in patients ranging between 5 and 42 years of age [83], others have demonstrated a lower efficacy of the drug [84,85]. Comparing olanzapine (average dose 7.9 mg/day) to haloperidol (average dose 1.4 mg/kg) in 12 children with ASD, results reported that 83% of patients treated with olanzapine versus 50% of those treated with haloperidol were considered responders to therapy [86]. The only randomized double-blind study lasted 8 weeks and, although carried out only on 11 young subjects, reported an improvement in clinical symptoms [87].

##### Paliperidone

Paliperidone seems to be effective in the treatment of individuals with ASD, although studies are still limited. Over the years, several case studies have highlighted significant improvements in irritability and aggression treated with intramuscular (IM) and oral paliperidone (6 to 12 mg/day) without significant adverse effects, except for slight weight loss and increasing appetite [88,89]. Moreover, an open-label study conducted on 25 ASD patients, aged between 12 and 21 years (average age, 15 years), demonstrated an excellent response rate to oral paliperidone (dose 3–12 mg/day) in the treatment of irritability, this time in the presence of adverse effects such as mild to moderate extrapyramidal symptoms, weight gain, and increased prolactin [88].

##### Clozapine

Several studies have demonstrated clozapine’s good tolerability and efficacy in the management of severe aggression and irritability in patients with ASD, although double-blind controlled studies are lacking. A first case report with clozapine (dose 100 mg/day) highlighted a marked improvement in hyperactivity, attenuated affectivity, language, and abilities following treatment with 100 mg/day clozapine [90], while other subsequent case reports have highlighted that treatment with a dose ranging from 200 to 475 mg/day in ASD subjects has led to marked improvements in destructive behavior, aggression towards others and self-harm, as well as a reduction in ritual behavior and an improvement in social commitment. In these cases, adverse effects were minimal [91,92,93]. Finally, a retrospective analysis found that treatment with clozapine leads to a decrease in aggression and in the number of psychotropic drugs needed to manage behavioral symptoms. Clozapine was overall well tolerated, although constipation and weight gain were often present; furthermore, one case of metabolic syndrome and one case of sinus tachycardia have been reported [94].

##### Lurasidone

To date, the effect of lurasidone on subjects with ASD has not yet been demonstrated. A first case report has shown good efficacy in the treatment of irritability, aggression, and impulsivity in the absence of significant adverse effects using 30 mg/day lurasidone [95]. To verify these promising data, a first randomized double-blind placebo-controlled study was carried out on 150 subjects with ASD, aged between 6 and 17 years. Lurasidone (dose 20–60 mg/day) did not show statistically significant efficacy for the treatment of irritability [52]. Further case reports in the literature have shown the beneficial effects of the drug [96].

##### Summary

While only risperidone and aripiprazole received FDA approval for ASD treatment, showing good efficacy in improving irritability, aggressiveness, nervousness, and anxiety symptoms, the literature reports controversial results on the efficacy of other antipsychotics [44,45,46,47]. To the best of our knowledge, atypical antipsychotics such as olanzapine, paliperidone, lurasidone, ziprasidone, clozapine, and quetiapine have been employed in irritability and impulsivity treatment, sometimes with good efficacy [73,74,75,76,77,78,79,80,81,82,83,84,85,86,87,88,89,90,91,92,93,94,95,96]. Finally, among typical antipsychotics, haloperidol seemed to improve maladaptive behaviors, irritability, emotional lability, feelings of anger, poor collaboration and learning, and the psychosocial environment [56,57].

### 3.2. Mood Stabilizers

Although mood stabilizers are among the most widely used drugs for the treatment of multiple psychiatric disorders, their use in subjects suffering from ASD is still debated. To date, results from the limited available literature have been encouraging; however, there is a need to perform further placebo-controlled studies to evaluate the tolerability and effectiveness of mood stabilizers and anti-epileptics as treatments for specific symptoms [97].

#### 3.2.1. Valproic Acid

Sodium valproate has been the most widely studied anti-epileptic drug with mood-stabilizing properties. A first study conducted on three children with ASD and EEG anomalies aged between 3 and 5 years old and treated with valproic acid (dose 300–450 mg/day) highlighted an improvement in language, skills, and social interactions, repetitive and maladaptive behaviors, maintenance of eye contact, and imaginative activities [98]. These interesting data were confirmed by subsequent case reports [99]. In a retrospective study, 14 ASD patients, with or without EEG abnormalities or seizures, received sodium valproate (dose 125–2500 mg/day; mean blood level approximately 75 mcg/mL). Ten subjects (71%) showed a substantial improvement in aggression, temper tantrums, and mood lability [100]. Considering double-blind randomized and placebo-controlled studies, valproic acid use (dose 20 mg/kg/day; mean blood level 76 mcg/mL) was associated with the appearance of slurred speech and mild cognitive slowing without any other improvement in the clinical picture of ASD [101], except for significant improvement in irritability [102]. Although valproate is generally well tolerated, some adverse effects, such as activation, rash, sedation, nausea, vomiting, and weight gain, have been reported in these studies [101,102]. Moreover, valproic acid should not be considered a first-choice drug for women of childbearing age, given the increased risk of fetal malformations in the case of pregnancy and the risk of developing polycystic ovary syndrome [51]. Nevertheless, new guidelines also suggest caution in prescribing valproic acid to autistic males during the fertility period [103].

#### 3.2.2. Lamotrigine

This drug has been poorly studied in the treatment of subjects with ASD. Indeed, there is a single randomized double-blind and placebo-controlled study, lasting 8 weeks, which evaluated the effectiveness of therapy with lamotrigine (dose 5 mg/kg/day) on a sample of 28 children with ASD, aged between 3 and 11 years. This study showed no significant differences between subjects treated with lamotrigine and placebo [104,105]. Further studies are needed.

#### 3.2.3. Levetiracetam

Studies on the effectiveness of levetiracetam in the treatment of patients with ASD have led to conflicting results. Indeed, in a first open-label study conducted on 10 ASD subjects, aged between 4 and 10 years, up to 13–54 mg/day of levetiracetam improved attention, hyperactivity, emotional lability, and aggressive behaviors only in 6 boys who had never undergone psychoactive therapy [106]. Moreover, in the only randomized double-blind study with a placebo conducted on 20 children with ASD, aged 5–17 years, higher levetiracetam dosages (mean dose 862.50 ± 279.19 mg/day) were not effective in treating ASD aggression and mood instability but seemed instead to possibly increase aggressive behaviors [107].

#### 3.2.4. Oxcarbazepine

Unfortunately, no double-blind, placebo-controlled clinical trials have been conducted to evaluate the effectiveness of this drug in ASD. The only significant retrospective study present in the literature analyzed clinical cases involving 30 subjects suffering from ASD, aged between 5 and 21 years, treated with oxcarbazepine with dosages ranging from 600 to 1800 mg/day. The results reported improving aggression, sleeping problems, attention, and cooperation of these patients, while important adverse effects were reported in approximately a quarter of patients, including hyponatremia, generalized epileptic seizures, allergy, and worsening irritability [108].

#### 3.2.5. Topiramate

A first open retrospective study conducted on 15 adolescent and childhood ASD patients demonstrated that topiramate (dose 100–400 mg/day) was good in the inattention and hyperactivity treatment with mild adverse effects such as sedation, cognitive difficulties, and skin rashes [109]. These data were confirmed by subsequent open retrospective and double-blind placebo-controlled studies in which topiramate was reported to cause an improvement in hyperactivity, interpersonal behavior, irritability, anger, anxiety, and depression [110,111]. Noticeably, a double-blind placebo-controlled study was carried out on 40 children with ASD, aged between 4 and 12 years, treated with the combination of topiramate (dose 100 mg/day for weight < 30 kg and 200 mg/day for weight > 30 kg) and risperidone (dose 2 mg/day for weight < 40 kg and 3 mg/day for weight > 40 kg). This association showed reduced irritability, stereotyped behaviors, hyperactivity, and noncompliance more than risperidone alone, without noteworthy adverse effects [111].

#### 3.2.6. Lithium

ASD subjects often show comorbid mood disorders. In this regard, the most interesting study seems to be a retrospective review conducted on 30 clinical cases of ASD subjects not responding to antipsychotics, aged between 5 and 21 years, treated with lithium (blood level 0.70 mEq/L). Despite almost half of the sample experiencing side effects due to lithium, such as vomiting, tremors, fatigue, enuresis, and irritability, the authors reported an improvement in symptoms related to elevated mood, mania, hypersexuality, paranoia, and reduced need for sleep [112]. To date, a case report on two patients with ASD highlighted that lithium therapy ranging between 1000 and 1500 mg/day, with blood lithium levels ranging between 0.8 and 0.7 mEq/L, led to the regression of catatonia and the stabilization of behavioral symptoms without significant adverse effects [113].

#### 3.2.7. Summary

Despite the literature on mood-stabilizer efficacy in ASD treatment still being scarce, the available evidence has demonstrated a regression in behavioral symptoms after their use [113]. It is worth noting that valproic acid seems to improve language skills and social interactions, repetitive and maladaptive behaviors, maintenance of eye contact, and imaginative activities [98].

### 3.3. Antidepressants

Several investigations suggest a role for 5-HT dysfunction in ASD and related pervasive developmental disorders [114]. Antidepressant medications, particularly selective serotonin-reuptake inhibitors (SSRIs), have been used in the treatment of ASD patients with controversial results [37,115]. Despite some studies showing noticeable improvements in compulsions, anxiety, depressive symptoms, and aggression, decreasing the severity of symptoms [116,117,118,119,120,121], others have reported hyper-activation and agitation [122,123]. Fluoxetine, sertraline, citalopram, escitalopram, and fluvoxamine are the most common SSRIs evaluated in ASD [124]. Moreover, serotonin-norepinephrine-reuptake inhibitors (SNRI) (trazodone and mirtazapine), tricyclic antidepressants (TCA) (clomipramine), and other drugs (buspirone) have also been tested for the treatment of ASD with variable results [125,126,127].

#### 3.3.1. Selective Serotonin-Reuptake Inhibitors (SSRI)

##### Fluoxetine

Results regarding the use of fluoxetine in patients with ASD did not show unambiguous results. Several randomized studies have reported limited improvements in a restricted range of symptoms, such as anxiety and repetitive behaviors [36,120,128]. It is worth noting that a first randomized study on six adult ASD patients treated with fluoxetine for 16 weeks reported inconsistent improvement in anxiety symptoms and repetitive behaviors [128]. A subsequent double-blind randomized clinical trial highlighted an improvement in repetitive behaviors without detecting any problems of safety or tolerability in 45 children or adolescents with ASD during 20-week treatment with fluoxetine (average dose 9.9 mg/day). However, no improvement was observed in other ASD symptoms [120]. These data have been confirmed by a more recent study [36]. However, a further randomized study performed on 158 young subjects with ASD failed to detect any significant improvement in repetitive behaviors [129]. More recently, two additional large-scale randomized studies involving 158 and 143 children and adolescents with ASD reported that the efficacy of fluoxetine, administered for 14 and 16 weeks, respectively, was comparable to or marginally greater than that of placebo [129,130,131]. We could infer that current literature regarding the use of fluoxetine in autistic patients is still controversial and debated [132].

##### Sertraline

Few studies have evaluated the efficacy and tolerability of sertraline in patients with ASD. A first small study lasting 4 weeks and carried out on a small sample of 9 adult ASD patients suggested that sertraline (at doses between 25 and 150 mg/day) improved self-harm and aggression in the presence of minimal adverse effects [116]. Subsequently, a further small open clinical study reported that 2–8 weeks of treatment with sertraline (25–50 mg) significantly reduced anxiety and irritability in 8 pediatric patients [122]. At the same time, a broader open-label study lasting 12 weeks with higher doses of sertraline (50–200 mg) confirmed the drug’s efficacy in reducing aggressive and repetitive behaviors in adult patients with ASD. The drug was well tolerated with minimal adverse events [62]. Regarding sertraline use in children, a more recent 6-month study showed no benefit in 58 autistic children aged 2 to 6 years old receiving this treatment [133]. Another double-blind randomized 6-month placebo-controlled clinical trial of low-dose sertraline in children (3–6 years) reported a lack of efficacy in language development or changes in molecular markers. These results indicate that sertraline may not be beneficial during childhood [134].

##### Citalopram

Despite a limited field of the literature revealing improvements in aggression, anxiety, repetitive behaviors, and irritability [135], a 12-year multicenter placebo-controlled study conducted on 149 children with ASD aged between 5 and 17 years old found no differences in global improvement of ASD patients (average dose, 16.5 mg per day) compared to the controls. Furthermore, a series of adverse effects, including hyperactivity, insomnia, impulsivity, repetitive behaviors, diarrhea, and epileptic seizures, occur. From this perspective, citalopram has been considered scarcely effective for the treatment of patients with ASD [136].

##### Escitalopram

Studies evaluating the efficacy and adverse effects of escitalopram in subjects with ASD are still scarce. A first open-label study lasting 10 weeks with 20 mg/day of escitalopram reported a reduction of impulsivity and positive effects in general psychosocial functioning. However, a large number of patients experienced side effects such as hyperactivity, aggression, or irritability that led to early termination of treatment [121]. These results were also confirmed by a subsequent study [137].

##### Fluvoxamine

Clinical results with fluvoxamine are conflicting. The first double-blind study, lasting 12 weeks, was conducted on 30 patients with ASD, aged between 18 and 53 years old, treated with an average dose of up to 276.7 mg/day of fluvoxamine. Data reported some clinical benefits in repetitive thoughts, maladaptive behaviors, aggression, and language, with side effects mostly limited to nausea and sedation [117]. Conversely, further studies reported no clinical benefits from fluvoxamine treatment, while adverse effects such as insomnia, hyperactivity, aggression, agitation, irritability, impulsiveness, reduction concentration, and alterations in eating patterns have been found [117,138]. An open study on 18 ASD patients, aged between 7 and 18 years old, with comorbid anxiety and compulsive symptoms found that 83% did not show significant improvements in ASD or anxiety symptoms after 10 weeks of treatment with a low dose of fluvoxamine (1.5 mg/kg/day) [138]. Finally, a 12-week randomized double-blind placebo-controlled crossover study performed on 18 children with ASD reported an improvement in behavioral assessments in 10 patients [139].

##### Paroxetine

To date, paroxetine is the least studied SSRI, presenting less evidence for ASD patient treatment. Indeed, only a few case reports presented paroxetine as being fairly effective in reducing self-injurious behaviors and irritability, respectively, with dosages up to 20 mg/day and 10 mg/day [118,140]. Agitation and insomnia were the most reported side effects. Moreover, paroxetine is the SSRI associated with the greatest risk of suicide in these patients [118,140].

##### Summary

As SSRIs are the most employed in the treatment of anxiety symptoms [120,122,128,135] associated with ASD, the efficacy of this class of drug is still unclear. While sertraline showed a reduction in irritability and aggressiveness [62,122], citalopram, escitalopram, and fluvoxamine seemed to improve these symptoms in some cases [136,137,138]. For this reason, further studies are needed.

#### 3.3.2. Serotonin/Norepinephrine-Reuptake Inhibitors (SNRI)

##### Mirtazapine

Mirtazapine, belonging to the class of SNRI (serotonin/norepinephrine-reuptake inhibitor), has been considered to be useful for ASD treatment [141]. An open-label examination conducted on 26 subjects aged ages 3.8 to 23.5 years old reported important results in aggression, self-harm, irritability, hyperactivity, anxiety, depression, and insomnia, but no improvement in the other fundamental characteristics of the disorder, with mirtazapine dosages ranging between 7.5 and 45 mg/day. Adverse effects were mild and included gain in appetite and weight, irritability, and transient sedation [126]. Furthermore, an open-label study conducted on 10 subjects with ASD aged between 5 and 16 years revealed an 80% response rate on inappropriate sexual behaviors [142].

##### Venlafaxine

Despite the literature on this topic being limited, venlafaxine proved to be quite promising in the treatment of ASD symptoms. A case report documented improvements in self-harm and hyperactivity with a venlafaxine dose up to 18.75 mg/day in two 17-year-old boys and a 23-year-old [127]. Moreover, a retrospective review documented a satisfying response to venlafaxine with dosages ranging between 6.25 and 50 mg/day in repetitive behaviors and interests, social deficits, communication, inattention, and hyperactivity. The adverse effects were modest, including behavioral activation, inattention, polyuria, and nausea [143]. Finally, a more recent randomized double-blind study added venlafaxine 18.5 mg/day or placebo to the zuclopenthixol and/or clonazepam treatment in 13 autistic patients. No differences between the venlafaxine or placebo groups and no significant decrease in hyperactivity/noncompliance were observed during the study. The authors concluded that reduced cumulative doses of clonazepam and zuclopenthixol were required for the venlafaxine group [144].

##### Trazodone

To the best of our knowledge, the literature shows only case reports that have highlighted the efficacy of trazodone (150 mg/day) in determining a reduction in aggression and self-harm in some patients [125]. Conversely, several studies have highlighted trazodone’s effectiveness in the treatment of ASD subjects’ sleep disorders [145,146,147].

##### Summary

To the best of our knowledge, most studies on SNRIs have focused on the potential effects they may have on additional autistic symptoms such as anxiety, depression, and insomnia [126,145,146,147]. Moreover, venlafaxine has shown interesting results in improving social deficits, communication, inattention, and hyperactivity [143]

#### 3.3.3. Other Antidepressants

##### Buspirone

Buspirone is a serotonergic agent that has been studied for the treatment of irritability and anxiety in ASD. Some clinical cases have reported an effective reduction in hyperactivity and aggressive behaviors and an increase in performance in the absence of significant adverse effects [148]. The only prospective open-label study present in the literature recruited 22 patients with ASD aged between 6 and 17 years, showing that 16 of the 22 subjects had significant responses to buspirone for dosages ranging between 15 and 45 mg/day. Only one patient developed abnormal involuntary movements. From this perspective, authors suggest buspirone as a substantial alternative to antipsychotics, when not well tolerated, in the treatment of irritability and/or anxiety [149]. It is worth noting that in a more recent 10-week randomized double-blind parallel-group placebo-controlled study on 44 autistic outpatients aged 4 to 12 years, buspirone improved irritability, lethargy, social withdrawal, and hyperactivity, while it had no efficacy on stereotyped behavior and inappropriate language. Vomiting and headache were the most frequently reported side effects [150]. A further randomized study on 166 children with ASD aged 2–6 years receiving 2.5 or 5.0 mg of buspirone twice daily demonstrated this drug’s efficacy in reducing restrictive and repetitive behaviors in these subjects [151].

##### Clomipramine

The role of clomipramine in the treatment of subjects with ASD is still debated. The first clinical case of a 12-year-old patient with ASD treated with a dose of 75 mg/day documented a reduction in self-mutilation, irritability, and hypersensoriality [152]. This first study was followed by a case series confirming previous results [153], while several open studies demonstrated a reduction in motor disorders, compulsions, and repetitive thoughts and behaviors, as well as improvements in aggression and aspects of social relationships [154,155] at dosages up to 75 mg/day to 250 mg/day [156,157,158]. However, other studies did not prove clomipramine’s therapeutic effects in managing stereotypies, aggression, and hyperactivity in children [156,157]. Moreover, clomipramine seems to be followed by several adverse effects such as sleep disturbances, dry mouth, constipation, urinary retention, fatigue or lethargy, dystonia, depression, behavioral problems, increased aggression and irritability, prolongation of the cardiac QT interval, and severe tachycardia [156,158,159]. A subsequent study compared the effectiveness of clomipramine and haloperidol. Results revealed that haloperidol is superior at reducing ASD symptom severity, as well as irritability and hyperactivity [160]. At the same time, other studies reported clomipramine efficacy in autistic subjects reporting repetitive behaviors and insistence on sameness, especially when comorbid with obsessive–compulsive disorders [161,162].

### 3.4. Psychostimulants

According to several studies, ASD and ADHD share high comorbidity rates, ranging between 18 and 50% for children with ADHD with ASD symptoms [163,164,165], while, conversely, 40–70% of children with ASD present ADHD symptoms [166,167,168]. As the main treatment of patients with ADHD is represented by psychostimulant drugs, reducing hyperactivity and impulsivity in approximately 70–80% of patients [169], methylphenidate seems to be effective in reducing hyperactivity and impulsivity in 50% of children with ASD [170,171,172]. Either way, patients with ASD are not usually able to tolerate doses equivalent to those of patients with exclusively ADHD. For this reason, lower doses should therefore be evaluated (range of 2.5 to 10 mg^2^/day) to avoid the appearance of adverse effects, including irritability, stereotyped behaviors, and gastro-intestinal and sleep problems [169,173]. Finally, psychostimulants do not present beneficial effects on other symptoms such as irritability, social withdrawal, repetitive behaviors, or language disorders [173].

### 3.5. Alpha-2 Adrenergic Receptor Agonists

Alpha-2 adrenergic receptor agonists are used in ASD patients’ treatment of aggressive behavior, sleep disorders, and anxiety. Indeed, by inhibiting noradrenergic neurotransmission in the brainstem, these drugs lead to a decrease in hyperarousal, anxiety, and motor spasms.

#### 3.5.1. Clonidine

A first randomized double-blind study on ASD subjects aged 5–33 years old reported a 4-week clonidine treatment (dose 0.005 mg/kg/day) reducing hyperarousal behaviors and improving the social interactions in these patients [174]. Further studies highlighted a modest improvement in irritability and hyperactivity after treatment with clonidine [175] and important effects in reducing sleep onset latency, night waking, hyperactivity, and aggression in children with ASD [176]. No significant side effects were found in any of these studies [174,175].

#### 3.5.2. Guanfacine

Only a few studies have explored the effects of guanfacine, reporting that a dose of 3 mg/day in ASD patients causes a reduction of hyperactivity [177], impulsivity, and distractibility [178]. However, adverse effects, including drowsiness, fatigue, and decreased appetite, have been reported [177,178]. A more recent study on 53 boys with ASD aged 5–14 years reported important improvements also in stereotypic behaviors, inappropriate speech, and opposition at dosages ranging between 0.25 and 9 mg/day [179].

#### 3.5.3. Summary

Among alpha-2 adrenergic receptor agonists, clonidine and guanfacine presented a modest improvement in reducing hyperactivity, irritability, and aggressiveness in ASD [175,177].

### 3.6. Cholinesterase Inhibitors and NMDA-Receptor Antagonists

Some studies, however, have suggested that an alteration of excitatory–inhibitory neurotransmission at the level of some brain areas can play an important role in ASD pathophysiology. From this perspective, several drugs whose targets are glutamate and gamma amino butyric acid (GABA) have been examined [180,181,182].

#### 3.6.1. Memantine

This drug is an NMDA-receptor antagonist, generally used in the therapy of Alzheimer’s disease. A first 8-week open-label trial investigated the effects of memantine (dose up to 20 mg/day) in 14 subjects with PDD (pervasive development disorder) aged between 3 and 12 years, reporting significant improvements in memory, irritability, lethargy, stereotypies, hyperactivity, and language deficit [183]. From this perspective, further open studies evaluated this drug’s efficacy on ASD subjects. The largest trial recruited 151 ASD patients treated with memantine at dosages ranging between 2.5 and 30 mg/day. Data revealed a statistically significant improvement in behavior and language, although approximately one in ten patients had to discontinue memantine due to the onset of agitation [184]. A trial was conducted of a combination of 3 mg/day risperidone and 20 mg/day memantine, compared with a placebo for ten weeks. Subjects treated with risperidone and memantine showed a statistically significant reduction in behavior stereotypes, hyperactivity, and irritability. Adverse effects included pain, abdominal pain, nausea, decreased or increased appetite, dizziness, insomnia, sedation, and cutaneous rush [182].

#### 3.6.2. R-Baclofen

R-baclofen is an active enantiomer, a selective GABA-B agonist drug, and a centrally acting muscle relaxant often used for the treatment of spasticity during neurological diseases. Treatment with R-baclofen in ASD (dose 20–30 mg/day) showed an improvement in irritability, social function, and communication. The most frequent adverse events reported are agitation and irritability, for caused two subjects to interrupt the study, but fatigue, hyperactivity, insomnia, and diarrhea were also found [185].

#### 3.6.3. N-Acetylcysteine

Recent studies suggested the use of *N*-acetylcysteine combined with other drugs in several psychiatric disorders such as schizophrenia, bipolar disorder, obsessive–compulsive disorder, and pathological gambling [186,187,188,189]. A 12-week randomized placebo-controlled study recruiting 33 ASD children aged between 3 and 12 years treated with 2700 mg/day *N*-acetylcysteine reported a significant reduction in irritability, repetitive behaviors, and stereotypes. Among the adverse effects, only mild gastro-intestinal disorders were reported [190].

#### 3.6.4. D-Cycloserine

Interesting but controversial results came from the application of D-cycloserine in the treatment of ASD. A recent systematic review reported low evidence of this drug’s efficacy [191]. At the same time, D-cycloserine seemed to improve social and communication skills in autistic subjects [192]. It is worth noting that a randomized controlled trial on ASD patients ranging from 5 to 11 years old revealed that D-cycloserine administration for 22 weeks may enhance the durability of social skills training [192]. Moreover, this drug has been shown to be effective in improving stereotypes in children and young adolescents with ASD [193]. To the best of our knowledge, only a randomized controlled study has focused on D-cycloserine effects in older adolescents and young adults. Results reported that daily or weekly administration of the same dosages (50 mg) for 8 weeks, with 2 weeks follow-up after discontinuation, led to no differences in improving social behaviors in these patients [194].

#### 3.6.5. Summary

As cholinesterase inhibitors and NMDA-receptor antagonists showed significant improvements in irritability and social function cluster symptoms [183,184,185,190,192], further studies are needed to evaluate these encouraging results. See Table 2 for a summary of old strategies in autism treatment.

## 4. Other Therapeutic Strategies

Some authors have explored new therapeutic possibilities [195,196,197,198,199,200,201,202,203,204,205,206,207,208,209,210,211,212,213,214,215,216,217,218,219,220,221,222,223,224,225,226,227,228,229,230,231,232,233,234,235,236,237,238,239,240,241,242,243,244,245,246,247,248,249,250,251,252,253,254,255,256,257,258,259,260,261,262,263,264,265,266,267,268]. Focusing on the potential role of altered oxytocin levels and on the presence of enhanced inflammatory activity in ASD subjects, new molecules have been used in the treatment of autism with promising results that need further investigation [195,196,197,198,199,200,201,202,203,204,205,206,207,208,209,210,211,212,213,214,215,216,217,218,219]. It is worth noting that some authors have stressed the importance of gut-microbiota imbalance in the pathogenesis of the disorder [269,270,271,272,273,274,275,276,277,278,279,280,281,282,283].

### 4.1. Oxytocin

Oxytocin is a neuropeptide produced by the supra-optic and paraventricular nuclei of the hypothalamus. Due to its role in the structuring of mother–child and social bonding, oxytocin may be involved in specific attachment styles [195], improving interpersonal communication, affective and cognitive empathy, approach motivation, and social problem-solving [196,197,198]. Recent studies have reported the presence of lower oxytocin plasma levels in ASD subjects [199,200]. Focusing on its function, animal and human models have highlighted improved social behaviors through intranasal oxytocin spray [201,202,203,204,205,206]. From this perspective, 4-week clinical trials have shown a significant reduction in the activity of the amygdala following intranasal administration of oxytocin, resulting in improved social functioning and reduced repetitive activities; some studies have found a beneficial effect on social functioning in both children [207,208], and adults [209] improving eye gaze, emotion recognition and prosocial behaviors [209]. A recent meta-analysis evaluating the efficacy of oxytocin administration in ASD based on existing clinical trials and studies selected a total of 28 studies involving 726 ASD patients. Results demonstrated an improvement in social functioning, while non-social autistic domains remained unaltered by the oxytocin [206]. Intranasal oxytocin warrants further investigation as a promising therapeutic approach to address social deficits in ASD. Future research should focus on optimizing dosages, identifying subgroups that benefit most, and exploring long-term effects [204,205,206].

### 4.2. Anti-Inflammatory and Immunomodulatory Drugs

As recognized in recent literature, ASD pathogenesis underlines several causes. Recent evidence highlighted an important link between organic alterations and ASD, aiming to explore new therapeutic possibilities and innovative treatments [210]. Among them, studies reporting enhanced inflammatory activity seem to be particularly promising. Indeed, the disorder is associated with elevated pro-inflammatory cytokines, reduced levels of immune-regulatory cytokines, and an increase in circulating antibodies, leading to microglia and several brain alterations [210,211]. IL-1β, IL-6, IL-17, and TNF-α have major implications in ASD pathogenesis [212]. In particular, the over-expression of IL-6 [210] mediating abnormalities in brain development, such as dendritic spine length, shape, and distribution alterations, is frequently reported in neuroatypical subjects [213,214,215]. From this perspective, mesenchymal stem cells, differentiating in the mesenchymal lineage under appropriate conditions, may be included in new treatment strategies [216]. Indeed, these cells have shown immunosuppressive activity on the NK and T lymphocytes through the expression of prostaglandin type 2 (PGE2) and IL-10 [217], reducing inflammatory injuries on brain tissue [217]. Furthermore, they promote neuronal repair [217], enhancing synaptic plasticity and favoring the functional recovery of Purkinje cerebellar nuclei [218,219], which are frequently found to be reduced in autopsies of ASD patients [16]. Therefore, further studies are needed to better investigate the beneficial effect of reducing inflammatory dysregulation clearly linked to ASD onset.

### 4.3. Sulfurophane

The phytochemical sulfurophane has been tested because of its known anti-oxidant properties, the hypothesis being that ASD may be partly due to oxidative stress and mitochondrial dysfunction. Unfortunately, there are no univocal results in the literature. A controlled randomized double-blind study, which proposed sulfurophane treatment (dose 50–150 µmol/day) in ASD, showed an improvement in social interaction, abnormal behavior, verbal communication, irritability, and lethargy, but not stereotypes and hyperactivity. The authors hypothesized that the efficacy in decreasing ASD behavioral symptoms may be related to its anti-oxidant effects [220]. Subsequently, some studies have observed similar findings [221,222,223,224], but others have reported no significant clinical improvement in the behavioral outcome, although no side effects were recorded [225].

### 4.4. Spironolactone

Other interesting results were reported with spironolactone. Classified as a “potassium-sparing diuretic” and acting as a competitive aldosterone antagonist, it has anti-inflammatory, immunologic, and hormone-modifying properties. Currently, spironolactone is used in acne, hirsutism, and precocious puberty treatment. It is worth noting that a case study describing 4 weeks of daily spironolactone administration in an autistic adolescent reported significant improvement in the disorder’s symptoms, such as decreased irritability, lethargy, stereotypy, hyperactivity, and inappropriate speech, and receptive language skills [226].

### 4.5. Intravenous Immunoglobulin Treatment

Intravenous Immunoglobulin (IVIG) treatment has been tested as a potential treatment for ASD children with controversial results in terms of its efficacy in treating hyperactivity, irritability, and language deficits [227,228]. In a recent study, 14 autistic patients with immunological imbalances received 10 infusions of IVIG (1 g/kg) every 21 ± 7 days. The study assessed behavioral symptoms using various revealing improvements in social, language, and movement deficits [229]. Few studies have focused on cortico-steroid treatment efficacy in ASD. The literature has reported some case studies or reports of children receiving different dosages of prednisolone, from 0.5 mg/die to 2 mg/die, and progressively improving language, behavior skills, and motor stereotypes [230,231,232]. These findings may confirm the link between the disorder and elevated pro-inflammatory cytokines, reduced levels of immune-regulatory cytokines, and an increase in circulating antibodies leading to microglia and several brain alterations [203,204].

### 4.6. Celecoxib

Some authors have proposed a cyclooxygenase-2 inhibitor, celecoxib, as an adjunctive therapy in the treatment of ASD [233]. In a 10-week clinical trial, 40 ASD children were randomly assigned to receive either celecoxib in combination with risperidone or a placebo alongside risperidone. The results reported that the combination of risperidone and celecoxib was superior to risperidone alone in treating irritability, social withdrawal, and the stereotypical behavior of children with ASD [233].

### 4.7. Bumetanide

Bumetanide, a sodium-potassium chloride co-transporter inhibitor, has been proposed as a potential treatment for ASD due to its intrinsic chloride-related antagonistic effects linked to the GABA-ergic system [234]. Some randomized placebo-controlled 3-month trials conducted with the same equipment have shown that bumetanide reduces general ASD symptomatology without significant side effects [235,236]. Moreover, a single case report on a 10-year-old girl showed a marked clinical improvement in sensory behaviors, rigidity, and memory performance, confirmed by questionnaires and cognitive assessments [237]. From this perspective, a more recent 3-month open-label study in 6 children with severe ASD and intellectual disability showed parent-reported improvement in communication skills with bumetanide [238]. However, a recent double-blind placebo-controlled study in ASD children without severe intellectual disability reported no treatment benefit [239]. It is worth noting that a functional magnetic resonance imaging scan revealed a significant reduction in typical ASD overreacting amygdala and increased gaze time with biological stimuli or improved emotional facial perception after bumetanide administration [240,241]. To date, there is no conclusive evidence for the role of bumetanide in the treatment of ASD symptoms [242,243,244].

### 4.8. Balovaptan

The neuropeptide vasopressin has been implicated in the regulation of social behaviors, and its modulation has emerged as a therapeutic target for ASD. Balovaptan is a selective vasopressin V1a receptor antagonist. A 12-week phase II clinical trial focused on 223 ASD men treated with 4–10 mg/day of balovaptan, reporting no significant changes from baseline in the disorder’s core symptoms; however, clinically meaningful improvements were observed in the total score of the Vineland-II Adaptive Behavior Scale, particularly in the socialization and communication domains [245]. However, the follow-up studies did not confirm the results [246,247,248,249], and further studies are needed to better evaluate the potential use of the drug [250].

### 4.9. Pioglitazone

The link between ASD and the immune system has been discussed for several years [251,252]. Over recent decades, a growing field of research has focused on the central and peripheral immune system alteration and their possible implications in the structuring of the disorder, such as immune cell stimulation, autoantibody generation, cytokine/chemokine imbalance, and increased blood–brain barrier permeability [253,254,255]. Pioglitazone belongs to the group of thiazolidinediones acting via the peroxisome proliferator-activated receptor (PPAR)-γ [256]. Frequently employed as an antidiabetic agent offering additional cardiovascular protection and improvement of the lipid profile [257], these PPAR ligands also showed significant anti-inflammatory characteristics in psoriasis and asthma [258,259]. Moreover, the effects of neuropsychiatric disorders have also been studied [260]. A 12–16 week open-label study evaluating 25 subjects with ASD treated with pioglitazone at dosages ranging between 26 and 60 mg/day highlighted a significant decrease in hyperactivity, irritability, lethargy, and stereotypes; transient and self-limiting increases in white-blood-cell count, glucose level, and liver enzymes were reported among adverse effects [261]. A subsequent 10-week double-blind placebo-controlled study in 44 ASD patients already receiving risperidone combined with pioglitazone (30 mg/day) confirmed an improvement in hyperactivity, irritability, and lethargy in the absence of significant adverse effects [262].

### 4.10. Galantamine

Galantamine is a cholinesterase inhibitor, preventing the breakdown of acetylcholine and stimulating nicotinic cholinergic receptors [263]. Only a few studies have reported the use of galantamine in individuals with ASD. A case series observed increasing expressive language and communication with galantamine dosages ranging between 4 and 16 mg/day [264]. A subsequent 12-week, open-label, uncontrolled study in 13 children with ASD (mean age 8.8 years) reported improvements in social withdrawal, irritability, attention, and emotional lability [265]. Further studies highlighted significant improvement with galantamine in irritability, eye contact, hyperactivity, lethargy/social withdrawal, and inappropriate speech [266,267,268].

### 4.11. Microbiota-Transfer Therapy

Children with ASD often experience gastro-intestinal functional disorders, impacting their well-being. Emerging evidence suggests that a gut-microbiota imbalance may exacerbate core and gastro-intestinal symptoms [269]. Food selectivity and other eating disorders are separate yet common phenomena in children with ASD, observed in 45–89% of patients. Low fiber intake, along with high simple carbohydrates and saturated fats, can lead to a major shift in the gut microbiota [270]. This type of diet has been observed to be related to functional gastro-intestinal symptoms. A connection between these symptoms and major or behavioral difficulties in ASD has been previously reported, and the correlation at the microbial level may support this observation [271]. An exclusion diet, with the elimination of casein and gluten, is frequently followed by children with ASD (13% to 88% of families). There are no clear data on the beneficial effects of such diets, apart from those with proven food allergies and celiac disease [272,273]. Positive results have been observed in some patient groups; however, the studies were conducted in small groups or based on parental reporting questionnaires [274,275]. The exclusion diet led to an increase in the abundance of bacteria, which have been observed to be related to irritable bowel syndrome symptoms in neurotypical patients [276,277]. Probiotics appear to be the simplest intervention, with their exact mechanism of action remaining unclear between a simple improvement of dysbiosis or reduction of intestinal inflammation. While these drugs could lead to behavioral and affective improvement [278], prebiotics also appear to provide some improvement in gastro-intestinal and behavioral symptoms, both alone and in combination with probiotics [279,280]. Finally, microbiota-transfer therapy also led to improvements in functional gastro-intestinal symptoms, already observed in irritable bowel syndrome, and behavior, which appeared to persist long after the intervention [281,282]. The use of this new strategy in several psychiatric disorders showed promising results, including a reduction of anxiety and depression symptoms [283]. However, due to its invasiveness, microbiota-transfer therapy can be discouraging, especially in children [269].

### 4.12. Summary

As oxytocin intranasal spray was demonstrated to improve interpersonal communication, affective and cognitive empathy, approach motivation, and social problem-solving [195,196,197,198,199,200,201,202,203,204,205,206], several authors have considered the role of mesenchymal stem cells as a new treatment strategy related to the enhanced inflammatory activity in microglia and other signs of neuroinflammation in autistic subjects [210,211,212,213,214,215,216,217,218,219]. Moreover, a small field of literature has stressed the promising results of IVIG and cortico-steroid therapy in children or spironolattone and celecoxib in adults and other new strategies [220,221,222,223,224,225,226,227,228,229,230,231,232,233,234,235,236,237,238,239,240,241,242,243,244,245,246,247,248,249,250,251,252,253,254,255,256,257,258,259,260,261,262,263,264,265,266,267,268]. See Table 3 for a summary of new strategies in autism treatment.

## 5. Nonpharmacological Intervention in ASD: Cognitive–Behavioral Therapy, Music Therapy, and Pet Therapy

Although this review has focused on pharmacological treatments for ASD, in this section, we provide some insight into specific kinds of psychotherapeutic interventions that have gained attention in the field.

### 5.1. Cognitive–Behavioral Therapy and Psychotherapy

Given the high prevalence of ASD comorbidities with several psychiatric disorders, such as anxiety, mood, and obsessive–compulsive (OCD) disorders, a growing field of literature is focusing on the effectiveness of combining psychotherapy interventions with pharmacological therapies in these patients. Cognitive–behavioral therapy (CBT) is the best-known psychotherapy, lasting 5 to 20 sessions, typically once a week. During the treatment, patients learn to recognize and restructure negative cognitive schemas, either individually or in a group setting [284,285,286]. Some studies have confirmed the efficacy of CBT for the treatment of symptoms related to ASD, including anxiety, in ASD patients, while a limited number of studies have investigated its effectiveness on mood disorders and OCD in comorbidity [287,288,289]. Moreover, nonpharmacological strategies such as parent training seemed to be effective in the treatment of irritability. A recent meta-analysis highlighted that parent training reduced irritability in ASD subjects [290].

### 5.2. Music Therapy

In the framework of nonpharmacological interventions, auditory and sensory integration practices are gaining interest [291]. Music therapy is described as “a systematic process of intervention wherein the therapist helps the client to promote health, using music experiences and the relationships that develop through them as dynamic forces of change” [292,293]. Aiming to improve social interactions, the processing and integration of sensory stimuli, as well as creativity, music therapy favored the development of verbal communication, attention, and memory mechanisms in children with ASD [294]. Indeed, functional connectivity between the bilateral primary auditory cortex and subcortical and motor regions seems to increase in children who received music-based intervention [295]. Moreover, working in a team may encourage patients to develop social connections with others through an increase in joy, attention, eye contact, and turn-taking [296]. The literature has also confirmed the involvement of sensorimotor regions of the cerebellum and cerebro-cerebellar circuits in causing ASD motor control disability and repetitive and stereotyped behaviors [297,298,299]. From this perspective, music therapy aims to rewire sensorimotor activities, improving motor control and repetitive behaviors [300]. Indeed, after a biweekly rhythmic program, ASD subjects reported better motor skills [301]. In particular, rhythm interventions helped to control systemic pacing, ease repetitive behaviors, and reduce anxiety in ASD patients [302]. Furthermore, rhythmicity may have a potential impact on dyslexia disorder or, generally, in children with voice and language disorders [303]. Finally, children with proprioceptive deficits showed improved outcomes in listening and visual tasks and attention levels when they received proprioceptive rhythmic input [304,305,306].

### 5.3. Pet Therapy

A growing field of literature is focusing on the beneficial effect that interactions with animals can have on autistic subjects by helping them to better manage their emotions. Research evidence revealed that pet therapy contributes to improving the quality of life and social and work skills of ASD patients. Among various animal interactions, horse therapy seems to improve motor and postural skills [307], spontaneous verbalization [308], social cognition and receptive communication skills [309], cognitive function [310,311,312], as well as quality of life [313], reducing irritability, hyperactivity, and maladaptive behaviors [314]. At the same time, ASD children with severe social withdrawal showed improvement in the frequency of both verbal and nonverbal social behaviors after the introduction of a friendly dog [315]. Furthermore, interaction with dogs seems to reduce distraction and improve playful mood and awareness of the social environment [316,317]. ASD subjects reported beneficial effects also from cat companions. Indeed, cat therapy showed several positive outcomes, such as motivation for an active life [318], a better understanding of other people’s needs [319], learning about healthy eating through cat feeding [320], and fewer depressive symptoms [321].

## 6. Conclusions

Despite several studies reporting at least partial pharmacological efficacy of different drugs in ASD subjects, aripiprazole and risperidone remain the only psychopharmacological treatment with FDA approval for ASD [44,45,68]. Indeed, large, randomized trials on these drugs revealed a reduction in outbursts of anger, aggression, self-harm, and general regulation of disruptive behaviors [44,45,62,63,68]. To date, studies on other antipsychotics involved in ASD treatment are controversial and require wider sample sizes [73,74,75,76,77,78]. Several side effects, such as sedation, drowsiness, increased appetite, weight gain, hypersalivation, and the presence of EPS, are common [31,32]. To date, the ascertained safety and efficacy of other antipsychotics seem to remain lower than risperidone and aripiprazole [58,59], but more studies should be performed. Moreover, a small field of literature is encouraging the use of mood stabilizers to reduce swings in mood, irritability, impulsivity, anxiety, and anger associated with ASD features [110,111,112], even though their involvement in the treatment of the disorder is still debated. Finally, as several investigations suggested the role of serotonergic dysfunction in ASD [114], antidepressant medications, particularly SSRIs, have been tested [115] with controversial results [37]. At the same time, some antidepressive drugs seem to have promising results on specific symptoms [117,135,140].

Some authors have explored new therapeutic possibilities [195]. In this framework, Oxytocin intranasal spray has shown efficacy in improving interpersonal communication, affective and cognitive empathy, approach motivation, and social problem-solving [196,197,198]. Considering the role of enhanced inflammatory activity in microglia and other signs of neuroinflammation in autistic subjects [210,211,213,214,215], mesenchymal stem cells have been proposed as a new treatment strategy [216]. Through the immunosuppression of NK and T lymphocytes, they may promote neuronal repair and synaptic plasticity [217]. It should be noted that a small field of literature stressed the promising results of IVIG and cortico-steroid therapy in children or spironolattone and celecoxib in adults and other new strategies [220,226,230,231,232,233].

Obviously, the choice of therapy requires the evaluation of many factors. The heterogeneity in the disorder’s etiology may contribute to different clinical pictures manifesting as different deficits or impairments [322]. Indeed, ASD is considered to be the result of complex interactions among genetic, environmental, and immunological factors [323]. In light of new evidence, recent studies have searched for biomarkers implicated in the signaling pathways, such as transcription, translation, synaptic transmission, epigenetics, and immuno-inflammatory responses, aiming to find new therapeutic strategies [324]. While behavioral interventions, which were not the focus of this review, remain one of the principal intervention methods at present, being more effective when employed since early stages, a combination of pharmacological and nonpharmacological intervention may be suggested for improving irritability, anxiety, and depressive symptoms frequently deriving from the autistic core features [322,323,324].

**Figure 1:**
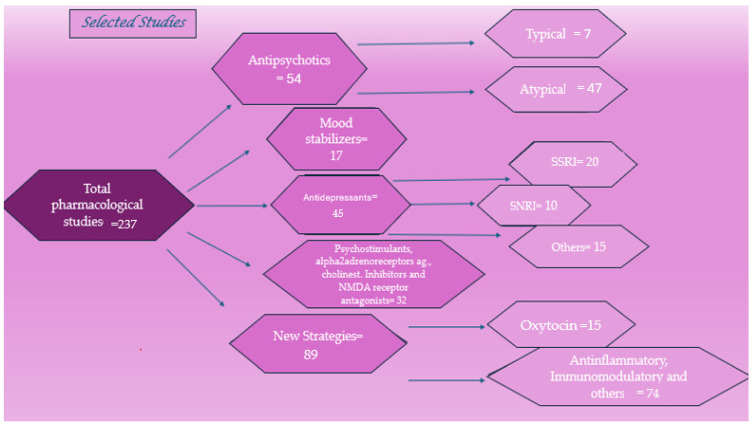
Selected pharmacological articles.

**Figure 2:**
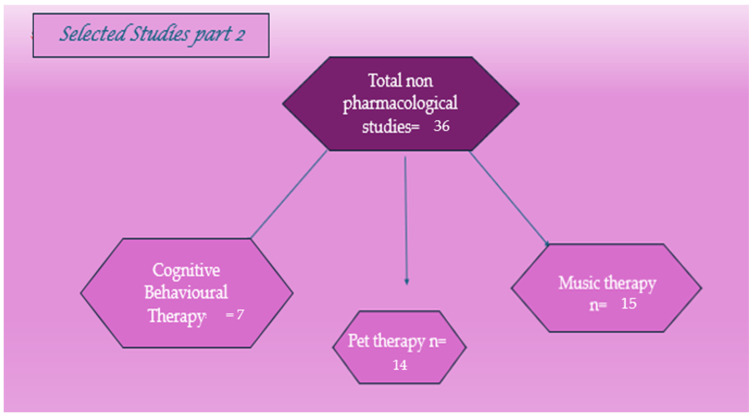
Selected non pharmacological articles.

Globally, the available literature highlighted that there is a lack of proper trials for evaluating the possible efficacy in ASD of several pharmacological treatments approved for other mental disorders, on which further studies should focus. On the other hand, new therapeutic targets identified based on biological studies on ASD demand to be accurately tested. It appears crucial to raise awareness among clinicians in the field on the importance of employing integrated therapeutic strategies, as well as to focus on personalizing as much as possible the treatment of ASD subjects, who often show poor responsivity to the available resources (See Figure 1 and Figure 2 for a summary of articles selected).

## Figures and Tables

**Table 1:** ASD pathogenesis causes.

ASD Pathogenesis Causes	Mechanism	Literatures
Genetic causes	More than 200 genes involved: - chromosomal rearrangements - de novo or inherited copy number variations - susceptibility genes across several chromosomes, including the *X*, *2q*, *7q*, and *15q*	[12,13,14,15]
Environmental causes	Intrauterine causes: - advanced maternal and paternal age - valproate intake - toxic chemical exposure - maternal diabetes - enhanced steroidogenic activity	[16,17]
Biochemical factors	- Enhanced inflammatory activity and microbiota alteration - Serotonin (5-HT) system dysregulation - Tryptophan metabolic route alteration - Disruption in glutamate excitatory and GABA inhibitory systems - Oxytocin dysregulation - Altered methylation activity - BDNF levels alteration	[18,19,20,21,22,23,24,25,26,27,28]

**Table 2:** Pharmacological therapies: old strategies.

Antipsychotics	Risperidone	* Approved by the FDA for the treatment of irritability cluster symptoms in ASD	[44,45]
	Aripiprazole	* Approved by the FDA for the treatment of disruptive behaviors in ASD	[46,47]
	Quetiapine	Could be useful for sleep disorders and aggression in ASD	[73,74,75,76,77]
	Ziprasidone	Improvement of aggression, agitation, and irritability in ASD	[78,79,80,81,82]
	Olanzapine	Efficacy in treating irritability, anger, anxiety, hyperactivity, social withdrawal, and use of language in ASD	[83,84,85,86,87]
	Haloperidol	Efficacy in reducing the severity of social withdrawal and stereotypies, maladaptive behaviors, irritability, emotional lability, feelings of anger, poor collaboration and in improving learning and psychosocial environment in ASD	[55,56,57]
	Pimozide	Could be useful in the treatment of sleep and excretion disorders in ASD	[60,61]
	Paliperidone	Improvements in irritability and aggression in ASD	[88,89]
	Clozapine	Improvement in hyperactivity, attenuated affectivity, language and abilities, destructive behavior, aggression towards others and self-harm, social commitment, and a reduction in ritual behavior in ASD	[90,91,92,93,94]
	Lurasidone	Efficacy in the treatment of irritability, aggression, and impulsivity in ASD	[95,96]
Mood stabilizers and anti-epileptics	Sodium valproate	improvement in language, skills, and social interactions, repetitive and maladaptive behaviors, maintenance of eye contact and imaginative activities in ASD	[98,99,100,101,102,103]
	Lamotrigine	No significant improvements in ASD	[104,105]
	Levetiracetam	Improved attention, hyperactivity, emotional lability, and aggressive behaviors in ASD	[106,107]
	Oxcarbazepine	Improving aggression, sleeping problems, attention, and cooperation in ASD	[108]
	Topiramate	Improvement in hyperactivity, interpersonal behavior, irritability, anger, anxiety, and depression in ASD	[109,110,111]
	Lithium	Improvement in symptoms related to elevated mood, mania, hypersexuality, paranoia, and reduced need for sleep in ASD	[112,113]
Antidepressants	Fluoxetine	Improvements in anxiety and repetitive behaviors in ASD	[128,129,130]
	Sertraline	promising in the treatment of self-harm, aggression, anxiety, irritability, aggressive and repetitive behaviors	[133,134]
	Citalopram	Improvements in aggression, anxiety, repetitive behaviors, and irritability in ASD	[135,136]
	Escitalopram	Reduction of impulsivity and positive effects in general psychosocial functioning in ASD	[137]
	Fluvoxamine	Improvements in repetitive thoughts, maladaptive behaviors, aggression, and language	[138,139]
	Paroxetine	Fairly effective in reducing self-injurious behaviors and irritability in ASD	[140]
	Buspirone	Effective reduction in hyperactivity and aggressive behaviors and increase in performance,	[148,149,150,151]
	Mirtazapine	Improvements in aggression, self-harm, irritability, hyperactivity, anxiety, depression, and insomnia. Good response in reducing inappropriate sexual behaviors	[141,142]
	Venlafaxine	Improvements in repetitive behaviors and interests, social deficits, communication, inattention, and hyperactivity	[143,144]
	Trazodone	Reduction in aggression and self-harm	[145,146,147]
	Clomipramine	Reduction in self-mutilation, irritability, hypersensoriality, motor disorders, compulsions, repetitive thoughts and behaviors, aggression and aspects of social relationships	[153,154,155,156,157,158,159,160,161,162]
Psychostimulants	Methylphenidate	Effective in reducing hyperactivity and impulsivity in ASD	[169,170,171,172]
Alpha-2 adrenergic receptor agonists	Clonidine	Reducing hyperarousal behaviors and improving social interactions in ASD	[174,175,176]
	Guanfacine	Reduction of hyperactivity, impulsivity, and distractibility in ASD	[177,179]
Cholinesterase inhibitors and NMDA-receptor antagonists	Memantine	Improvements in memory, irritability, lethargy, stereotypies, hyperactivity, and language deficit in ASD	[182,183,184]
	R-Baclofen	improvement in irritability, social function, and communication in ASD	[185]
	*N*-acetylcysteine	Reduction in irritability, repetitive behaviors, and stereotypes	[190]

* Approved by the FDA.

**Table 3:** New options.

Therapeutic Strategies	Beneficial Effects	Literatures
Oxytocin	Results demonstrated an improvement in social functioning	[201,202,203,204,205,206,207,208,209]
Anti-inflammatory and anti-immunomodulatory drugs	- Immunosuppressive activity on NK and T lymphocytes - reducing inflammatory injuries on brain tissue - promoting neuronal repair, enhancing synaptic plasticity, and favoring functional recovery of Purkinje cerebellar nuclei	[210,211,212,213,214,215,216,217,218,219]
Sulfurophane	improvement in social interaction, abnormal behavior, verbal communication, irritability, and lethargy, but not in stereotypies and hyperactivity	[220,221,222,223,224,225]
Spironolactone	Significant improvement in the disorder’s symptoms, such as irritability, lethargy, stereotypy, hyperactivity, and inappropriate speech decreased, and receptive language skills	[226]
Intravenous immunoglobulin	Efficacy in hyperactivity, irritability, and language deficit	[227,228,229,230,231,232]
Celecoxib	Improvement of irritability, social withdrawal, and stereotypy of children with ASD	[233]
Bumetanide	Improvement in communication skills	[234,235,236,237,238,239,240,241,242,243,244]
Balovaptan	Improvement in communication skills	[245,246,247,248,249,250]
Pioglitazone	Significant decrease in hyperactivity, irritability, lethargy, and stereotypes	[251,252,253,254,255,256,257,258,259,260,261,262]
Microbiota-transfer therapy	Reducing social and gastroenteric symptoms	[263,264,265,266,267,268]

## Data Availability

All data generated or analyzed during this study are included in this published article.
